# International Health Practices: A Multidisciplinary Approach to Therapeutic Mediations With an Artistic Medium Based on the Model of Play

**DOI:** 10.3389/fpsyg.2020.00254

**Published:** 2020-02-28

**Authors:** Anne Brun, Louis Brunet, Denis Cerclet, Antonie Masson, Magali Ravit, Jean-Pol Tassin, Silvia Zornig, Maria Clelia Zurlo, Tamara Guénoun, Sylvain Missonnier, Vincent Di Rocco, Lila Mitsopoulou, Eric Jacquet, Johan Jung, René Roussillon

**Affiliations:** ^1^CRPPC, Lumière University Lyon 2, Lyon, France; ^2^GREPP, Section Psychodynamique, UQAM, Montreal, QC, Canada; ^3^UMR 5600, Environnement, Ville, Société, Lumière University Lyon 2, Lyon, France; ^4^CRID, Interdisciplinaire Déviance et Pénalité, Université catholique de Louvain, Louvain-la-Neuve, Belgium; ^5^Neuroscience Paris-Seine UMR 8246 CNRS/U1130 Inserm/Sorbonne Université, Paris, France; ^6^Family and Child, Theory and Clinic, University Pontificale Catholic de Rio de Janeiro, Rio de Janeiro, Brazil; ^7^Dynamic Psychology Laboratory (PsyDy Lab), University of Naples Federico II, Naples, Italy; ^8^PCPP, Paris Descartes University, Paris, France

**Keywords:** creativity, play, qualitative evaluation, therapeutic mediations, sensory-motricity, symbolization, multidisciplinarity

## Abstract

This article, corresponding to a part of the restitution of a financed international research project between France, Brazil, Canada, Italy and Belgium, aims to offer a modelisation and qualitative evaluation of mediation care settings based on an original methodological tool that involves identifying the typical games at the foundations of creativity, following a multidisciplinary perspective. Therapeutic mediations are settings or devices organized around a “pliable medium,” often artistic, like painting, modeling, writing and theater, which are very widespread in institutional practices, both in France and abroad. The scientific objectives of this research consist in a multi-disciplinary exploration (anthropology, criminology, neuroscience, clinical psychology) of the process of creative symbolization understood as a process of transformation involving play. According to this orientation, play can be defined as a psychic process whereby a subjective experience can be explored with pleasure, and consequently symbolized and appropriated. Our fundamental and original hypothesis is that play is at the source of the creative process, conceived as a work of metabolization by the psyche of playful experiences during the different stages of life. The review of the understanding of play in psychoanalysis, anthropology, criminology and neuroscience emphasizes the richness of this model and the importance of reflecting on the typical games in the field of psychic care. A clinical example of treatment in a pictorial therapeutic mediation setting of a child with psychotic disorders makes it possible to identify a number of typical games as well as the modalities of interpretation of the therapists through play. These multidisciplinary studies lead to the presentation of a general table of typical games, and these first results highlight the richness of identifying typical games in clinical settings. Ultimately, the multidisciplinary approach shows the interest of the model of play in the evaluation of therapeutic mediation settings, with a convergence of the different disciplines emphasizing the pertinence of this model. The scientific impact of this research overlaps with its societal impact, through the development of innovative tools for evaluating therapeutic mediations, in order to take account of the evolution of the different forms of social expression of psychic suffering.

## Introduction

Therapeutic mediations are fundamental care settings in the different therapeutic practices that concern a large number of health-care institutions at all ages of life. These settings are organized around a “pliable medium” (Milner, 1955) that is often artistic, such as painting, modeling, writing, music or theater, and they are very widespread in the different healthcare centers in institutional practices, both in France and abroad. Generally speaking, these therapeutic settings concern a possible provision of care for forms of expression of psychic suffering, all the evidence showing that a large portion of its modes of expression are derived from the social register (delinquency, multiple antisocial acts, auto-aggressive behavior, social exclusion, criminality, etc.) or the somatic register (addictions). In this article, we consider the consequences of this evolution of contemporary clinical forms on the therapeutic settings marked by the cultural context, approached from the angle of art, creativity and care.

The aim of this article, corresponding to a part of the restitution of a financed international research project, is to offer a modelisation and qualitative evaluation of these care settings involving mediation. The methodological tool employed is the identification and modelisation of the typical games at the basis of creativity, according to a multidisciplinary perspective.

Multidisciplinarity aims at creating a disciplinary gap allowing us to test the consistency of our hypotheses but does not aim at a multidisciplinary reading of a clinical situation. The approach is not integrative but complementary with a double objective: it attempts at producing a general modeling of a practice which remains clinical, starting from the notion of play used as paradigm. This modeling aims at building assessment tools using the clinical method.

This international research consists in identifying in a new way and in evaluating the forms of games at the origin of the processes of symbolization in the psychic development of each subject, depending on the stages of life, and pathological expressions, as well as on the role of the model of play in the field of psychic care. It is a matter of isolating an international modelisation of artistic therapeutic mediations, based on the model of play, a constant feature of care practices with artistic mediations in different countries, going beyond the singularity and diversity of these practices of therapeutic mediations in the different countries taking part in this research, Brazil, Canada, Italy and Belgium.

The fundamental and original hypothesis that underlies these studies is that the exploration of the creative process, as a psychic process of transformation, involves play; the work of creation is thus conceived as a work of metabolization by the psyche of experiences of play throughout the different stages of life, a process that allows for a subjective appropriation of these experiences and, consequently, for a potential revival of the subject’s creativity. The choice of focusing this research on the model of play stems fundamentally from the importance in psychic life of play and the processes of integrating lived experience. From early childhood onward play serves to tame difficult or enigmatic situations by exploring them and subjecting them to a series of transformations, the central aim of which is to symbolize them and to endow them with a bonus of pleasure that facilitates their integration within subjectivity. This essential characteristic is maintained in different forms in many adolescent and adult modes of functioning where, beyond the manifest activity, the equivalents of a child’s play can be found in the depths of psychic activity, presenting the same characteristics, and sometimes even the same forms and the same functions in the integration of lived experiences. Evaluation with the help of the paradigm of play makes it possible, as we shall see from a clinical example, to construct models for following step by step the successes and vicissitudes of the integration of experiences lived within the context of therapeutic mediations in the depths of psychic functioning. Such a modelisation therefore implies examining the operators of play starting with objects that function as “*objeux*” (Fédida, 1978; Roussillon, 1991, 2013), which refer to the mediating objects used in play, derived from the concept of “pliable” medium (Milner, 1955; Roussillon, 1991, 2013), around which the settings of therapeutic mediations, based on art, are organized.

In this research work, clinical psychology is predominantly represented in France and abroad because therapeutic mediations within health practices are the direct object of its studies, but this new investigation necessitates the intersection of this field with other disciplines, each of which is enriched by the contributions of the others. The specificity of the methodology employed thus concerns a multidisciplinary exploration of the role of play in the process of creative symbolization at work in therapeutic mediation settings. Three disciplinary fields other than clinical psychology will thus be examined. First, anthropology, interested in games in different social situations marked by change, and examines how, starting from an “instinct for communication,” individuals become engaged with their bodies in a process and in shared action. Second, criminology makes it possible to identify the perverse games, of seduction and fascination, of criminals. Finally, the question facing neuroscience is whether it is possible to identify the functioning of the brain in the process of playing.

## The Positioning of Research on Therapeutic Mediations and Play in Relation to the State of Art

This project is absolutely original in relation to the state of art which includes studies on play in the different disciplinary fields, in particular, anthropology and psychology, studies on the creative process in the analysis of works of art, and studies on therapeutic mediations, but no overall research linking these different questions. The perspective of this article consists in linking the operator of play with the process of creation for interdisciplinary purposes, and in identifying as a common point the organizers of play in artistic therapeutic mediations, with the aim of developing an international model of these health practices based on play.

The introduction of play as a paradigm for a part of clinical practice is a major dimension in the work of Winnicott, who shows how learning to play helps to transform the most painful situations into situations that are “ready for symbolizing” (“*bonnes à symbolizer*”). From this perspective, Roussillon (2008) has shown that a part of the treatment process, particularly in its most crucial moments, can be modelised as revived forms of certain types of games from childhood or from early childhood, for example, peek-a-boo, games of absence/presence, the mirror game, etc. A part of clinical symptomatology may be understood as the result of “interrupted” forms of these typical games which it is a matter of rekindling during the therapeutic process. R. Roussillon has shown that the paradigm of play helps to bring pleasure to situations where the first experience failed to produce any, and to include the partner, the other subject: it is necessary, therefore, to rediscover the traces of the play that did not take place historically and to re-establish it in the different therapeutic settings. He thus lists three types of play experiences:

- “autosubjective” games that promote the transition from autosensoriality, which refers neither to an external object nor to an internal object, to autoerotisms that imply the representation of an absent object;
- “intersubjective” games that help the child to gain access to intersubjectivity and to construct his transitionality;
- “intrasubjective” games, that is to say the creative experiments that the child makes alone, even if the object is nearby, thereby helping him to establish intrapsychic differentiations in a deeper way and leading to the *capacity to be alone in the presence of the other* (Winnicott, 1958).

An initial step consists in distinguishing between manifest play and latent play; a modelisation based on the model of play takes into account the question of the articulation of the manifest aspects of play and its latent aspects, the evaluation of the latter implying a theory of symbolization based on what is involved in the play, given that the fundamental psychic issue becomes increasingly complex during the different stages of life. According to this perspective, Roussillon (2008) has identified several forms of play, each of which corresponds to stages of the work: for example, the games of presence/absence already mentioned, games of construction/deconstruction, of fullness/emptiness, etc.

A large number of “typical games” can be enumerated, which have their place within temporality and historicity. The “typical games” that children enjoy, even if they present invariant forms over the course of life, are not in fact the same, or do not take on the same forms during the course of the subject’s development and of the degree of increasing complexity of psychic life. This attempt to offer a modelisation is valuable because it makes it possible to follow one and the same basic issue as it is elaborated progressively over time, and also to identify the typical forms that follow each other during this elaboration. Further, the degree of psychic complexity acquired by the issue considered can be evaluated. From one form of game to another, the change or progression in the psychic elaboration can thus be evaluated.

The question discussed in this article concerns the transposition of this model of play to settings of artistic therapeutic mediations.

The utilization of play and its evaluation in child therapies with a wide range of people who are suffering mentally have of course also been the object of many research studies in Anglo- Saxon countries, drawing on diverse theoretical references. Very often it is a group approach to play that is advocated. In this area, if the American psychoanalysts Slavson (1943) with “Play Group Therapy” and Schiffer (1969) were pioneers in this respect, more recent research attests to the great diversity of the settings and groups of people concerned, without, however, claiming to provide an overall modelisation.

To illustrate this diversity, let us cite, by way of example, Bell et al. (1989) who sought to evaluate a method called “Development Play Therapy,” whose objective is the secondary prevention of the effects of multiple deficiencies and deprivations on children in early childhood. Particular attention is given to the therapeutic relationship, to the setting of non-directive play, and to the utilization of diverse developmental settings. Even more centerd on the treatment of severe traumas, the collective work of Nicholson et al. (2010) shows the importance of group therapies through play and creation in the treatment of mistreated and abused children placed in specialized institutions. The contributions link up psychoanalytic theory and practices with the study of art and literature. Another example can be given, with reference this time to attachment theory, with the research of Simeone-Russel (2011) who, in connection with a population of children with ASD, mainstreamed in a kindergarten setting, studies the therapeutic impact of the “Theraplay” group, a sort of play therapy called “attachment-based play,” for helping children build better attachment relationships with others.

However, it’s also important to note that behavioral approaches based on skills training programs are currently starting to integrate play activities into their methods, particularly in the field of autism spectrum disorder. The Early Start Denver Model (ESDM) intervention program (Rogers and Dawson, 2010; Dawson et al., 2012), i.e., a program developed for intervention with children with autism spectrum disorder and derived from classic behavior conditioning methods, introduced playing activities as learning tools and motivational support. Indeed, the therapeutic work within the ESDM is based on proposing specific playing activities to the child aiming to promote learning skills determined by therapeutic purposes. Nevertheless, the ESDM differs from the model of play proposed in the present article. Indeed, whilst the playing activities proposed within the Denver model aims to support the acquisition of specific skills, the present model proposes playing activities as organizers of a process in which there aren’t *a priori* learning objectives that are evaluated after the intervention. Therefore, within the suggested framework, the playing activities aim to propose an experience of resuming early intersubjective context and its typical plays, allowing to access primitive experiences that have been interrupted in the early psychological, cognitive and affective development. Consequently, the present study aims to explore the role of playing activities and mediating objects (whose function refers to the concept of malleable medium) as well as to evaluate the early development of symbolization processes.

In all these studies, as in many others, it seems that play is capable of stemming morbid processes or of triggering salutary transformations. It is noticeable that even when the reference is not specifically psychoanalytic, the question of the place of play in the therapeutic relationship and in relationships between peers remains central.

We therefore see the paradigm of play as an organizing model of intersubjectivity and of the symbolization of experiences. It is at the crossroads of varied epistemological models explored by the following transdisciplinary perspective.

## Transdisciplinary Perspective on Play

### Anthropology and Play

Anthropology has attributed special importance to play, which it has likened to ritual, as explored by Huizinga (1938), ascribing it with a structuring role for society (Caillois, 1957; Roberts and Sutton-Smith, 1962). The question that is often discussed is that of a setting that would provide play with a specific space, thereby distinguishing it from daily life (Goffman, 1974; Bateson, 1979; Copier, 2005; Juul, 2008; Consalvo, 2009). Play is also conceived as a process and initiation to the adaptability of behaviors (Sutton-Smith, 1997; Malaby, 2007). The role of anthropology can thus no longer be one of documenting the diversity of cultures. If the social sciences were born from the distinction between the individual and society and have distanced themselves from psychology, it is now obvious that the interest for social dynamics, as manifested concretely in the chains of gestures, usages, communications and exchanges carried by individuals, can only lead to a rapprochement of these disciplines.

In anthropology, the question of play can be found both in art, sport and rituals and, more recently, in behaviors linked to “self-intensity” as well as to the complexity of daily life that is studded with situations that are not unified or focalized. This heterogeneity, linked to the relative opacity of each of the relations and to the superficiality and fugacity of exchanges, induces a reinforcement of the skills of attention and invention. The individual becomes a creator of himself and of his environment. Breaking with the idea of a volitional subject and of a “material reality,” we are entering a world that emerges from relationships. If creation depends on intention and intelligence, and confers order on what might seem to be indistinct, the notion of process refers to another way of thinking about time, space, society and art that is more anthropological than theological. Process is marked by the relational dimension, which should be understood as a reciprocal stimulus. The character of interdependence and correlation that unites the participants in the process is primordial. Everything is marked by continual movement, rapprochement and distancing, changes of rhythm.

Approaches to creation in terms of process are based on the plasticity of the self and the environment. Neither humans nor non-humans are constrained by any pre-established role. In art, mediation and play everything becomes possible. In the theater, for example, we have been witnessing for several decades a “crisis of the character” (Abirached, 1994). While theater had sought for a long time to be reality, through mimesis, Brecht (1963) insisted that the spectator and the actor must remain lucid. Henceforth theater became an act in itself, inventing a space-time of bodies in movement. With Sollers (1968), we might say that “theater is the place where thought has to find its body” (p. 9) so that it does not take the body as the theater of its confrontations. Artaud and Grotowski made theater a body exercise, as is mediation through the theories of care and play, when it is sustained by a body in action (Andrieu, 2007). Thought is no longer conscious but emerges from the externality of the body, from relationship and acts.

The theorization of ritualities (Turner, 1969; Bateson, 1979) and performance (Turner and Bruner, 1986; Schechner, 2013) called into question liminality and the frame, which require the existence of both a space of complicity, in which fiction can become truly real, and a frontier, which, rather than a line, is a province that makes it possible to seize the right moment to enter or leave the performance. In street theater, the scenic space is revealed by gestures. The entrance of passers-by into the space of play occurs progressively; they reveal themselves as spectators immediately. The actor enters gradually into his performance and is only really in it when he makes his entry. The tacit pact is valid both for art, mediation and play. It can only be established when the entry is successful and the participants, as a whole, start to invent a specific and fragile gestural space-time which can manifest itself very profoundly in such a dynamic of bodies. The social process engages the emotions, memory, a sensitive relationship to the environment, cognition and all the bodies in such a way as to combine them in the accomplishment of an action which, here, may contribute to revealing an individual or a situation. Play is also thought of as a process and initiation to the adaptability of behaviors (Sutton-Smith, 1997; Malaby, 2007). But play seems above to be studied at the level of its relations to ritual and rarely at the level of its therapeutic dimension.

### Criminology and Play

In the field of criminology we can observe more specifically the emergence of games that assume extreme forms, ranging from the most diverse antisocial acts to the most pernicious forms, which seem to be a sort of “playing” with life. The metapsychological foundations to which we shall refer highlight the terror of subjects who tend to escape it by resorting to behavior and action. If it seems impossible here to synthesize the various works which relate to the expression of violent behavior, it is clear that these clinical phenomena were initially understood as deficits (deficiency in representation, intolerance to frustration) in opposition to the usual functioning of thought processes. Moreover, these understandings addressed questions of transference and language in a way that ignored the problem of representation. Balier (1988, 1996, 2005) has the merit to have offered a psychoanalytic perspective on violent patients followed in detention. He thus gave an essential place to intersubjective modalities (psychodrama, co-therapy, therapeutic groups) allowing to question the “language” of violence in its most original aspects.

The concept of “play” permit to understand and clinically work with psychic processes underlying violent radicalization and terrorism such as psychic reversal processes aimed at attaining a form of control over dreadful and poorly represented anxieties. By reversing helplessness anxieties, by becoming the one controlling the fear and the terror in the other, the violent being seeks to acquire a better control over a past traumatic experience. These reversals are well documented in the development of violent radicalized “lone wolves” terrorists (Brunet, 2019) but also in many violent behaviors. In the field of criminality we can also identify in games of seduction and/or seduction-fascination, coupled with games that have a perverse coloring. Sexual acting out (rapes, pedophilia, unwanted sexual touching) relate to degenerate forms (in the sense of having lost their generative power of symbolization) of play with the body and to constraint. These perverse sexual games involve experiences of portraying pleasure and the impossibility of gaining access to it. In contrast to this degradation of the possibility of play, clinical and expertise-based practices try to re- establish subjective games where all possibility of play had come to a standstill. This is where the real interest of mediation practices and the symbolic effect of being open to the words and affects of the person being evaluated lie.

Even in acts of great violence, it is sometimes possible to detect the imprint of a message and a demand for a response from the object (Brunet, 2017), a response that could have nothing but a mirror function, a function of instinctual drive satisfaction, but could also sometimes have a containing function (Brunet, 2010). The questions raised implicitly are as follows: “What can you do with this violence? Can you do something with it for me? Can you survive it?” Sometimes the drive cannot be properly “psychisized” (or invested psychically), but the subject nonetheless hopes in a confused way for a response from the object to this quantity that is not easily representable.

The study of individual or group violence shows how some individuals are able to commit acts of great violence with a minimum of guilt. Several studies have highlighted the implementation of a process of desymbolization that serves to remove the inhibition against killing. Symbolization is a dynamic rather than a static and stable process which may follow a progressive or regressive path depending on the circumstances, depending on whether the psychic apparatus has to protect itself against a trauma, as well as on the object’s response and the psychic functions that it can bring into play. Certain acts of violence may be characterized by a logic of evacuation, but others may comprise a significant symbolizing potential. In this context, the study of the vicissitudes of the symbolizing contribution of play not only affords us a better understanding of the process leading to violence, but opens out onto the therapeutic dimension that facilitates a resumption of the processes of subjective appropriation.

Among the different forms of possible therapies, therapeutic mediations in clinical situations of violent acting out seem particularly appropriate, and are in part based on forms of symbolization through play. Starting from the assumption that the difficulty criminals have in recognizing themselves in their acts is linked to a sensation of being cut off from their own bodies, to a disorganization of sensoriality, marked by catastrophic experiences such as experiences of penetration, explosion, mutilation and dismantling, it is crucial to offer these criminal inmates treatment approaches that involve not so much a search for well remembered recollections, which proves impossible in these clinical situations, but rather sensations, reminiscences that have their origin in the sensory-motor register. A major finding in this clinical field is that early experiences cannot be recalled because they cannot constitute themselves as memories; they remain linked to bodily states and sensations and hark back to early traumatic experiences. From this point of view, mediations practised with criminal patients, such as olfactory sensory mediation (Leca and Brun, 2012) or pictorial mediation (Garnier and Brun, 2016), reactualize games of sensory exploration based on smells or sensations arising from the medium of painting. Starting from modalities of sensory exploration of the pliable medium in the form of play, therapeutic mediations help incarcerated criminal patients to reactualize, in the form of hallucinated sensations, catastrophic sensory-motor experiences in the nature of primitive agonies, to defuse their dangerousness through the playful aspect of the group, and to reactualize a subjective appropriation of these catastrophic sensory experiences. It is the transference of the sensory-motricity of the patients, associated with experiences of being in tune with the therapists in playful ways such as theatralizations, that enables them to evolve from this dismantled and destructive sensoriality toward a sensorial composition, toward a *mantèlement*,^1^ a reorganization of the sensory-motor register in relationship to the pliable medium. This process involves the task of bringing together sensory islets, an experience of sensory integration, and a subjective appropriation of this sensoriality, all of which requires a playful relationship to the group, intersubjective games, and a resumption of dream activity.

Another form of mediation, the group mediation device using photos (Photolanguage) helps to remobilize visual space. It enters into resonance with the visual perceptual traces left by the criminal scene which are of a kind that are unthinkable (Ravit, 2016). The mediation thus give fresh impetus to what has remained “unaccomplished” or at a standstill in the instinctual drive process. This approach helps to stimulate the “sensory transmodality,” that is, to remodel associatively the conjunction of several sensory modalities in a form of “sensory intersubjective play.” Group associations to photos bring into play other sensory experiences concerning the body (hearing, touch, smells, taste, etc.) thereby providing form and density (subjective and intersubjective) where patients seem individually overwhelmed by experiences of terror and imminent death (Ravit, 2019). In this way, photographic visual perception offers a platform for the association of qualities and “sensory entries” around which an event is accomplished, a story is played out, in a different way than in a succession of traumatic states, which, in essence, are uncompleted.

A combined approach of criminology and psychology thus seems particularly heuristic with regard to the question of the model of play in therapeutic mediations.

### Neuroscience and Play

Neuroscience has paid little attention specifically to the question of play as such or of play in children. Panksepp (2007, 2011), who has studied “affective neuroscience” in relation to the brain of mammals, is interested in play presented as a specific “emotional system” involved in the organization of social behaviors in man. He recommends the use of “social play” in the treatment of HDAD. But the majority of research studies in neuroscience concerning play consider it from the angle of addictions by focusing on the reward circuitry in addictions to gambling, to “pathological games.”

It is nonetheless possible to roll out studies on the “playing” brain, that is, on the cerebral activity of the brain of a subject who is playing, and to show that this activity liberates neuromodulators that are indispensable for the cognitive processing of information in the central nervous system that is necessary for brain functioning and its development. In the context of this research, J. P. Tassin has put forward neuroscientific hypotheses on the “playing” brain, while distancing himself from studies linked to “pathological games.” Indeed pathological play has nothing to do with the subject we are concerned with here, in other words, the question of what play – in particular that of the young child – can represent in mental development. The studies of Roussillon (2007) indicate that play may be considered as facilitating the self-representation and assimilation of reality. It also makes it possible, in his view, to symbolize, represent and elaborate a traumatic situation. Concerning its mode of functioning, it seems that play can be motivated by a compulsion to repeat and, in a somewhat more complex way, that it permits an interaction with the transference and the counter transference.

The young child’s play plays a role in stimulating the establishment of brain functioning and its development. It seems, indeed, that there are two kinds of information processing in the central nervous system and that play facilitates the oscillations between these two modes of functioning (Tassin, 1989). The first mode, analogical, is fast and we do not have access to it. It can associate two events on the condition that they take place simultaneously. It is based on physical elements, both at the level of sensory and affective perceptions (lines, contrasts, forms, smells, noises, pains, caresses, pleasure, etc.) and at the level of the processing of this information as such (membrane channels, receptors, etc.). Thus, an individual’s analogical processing depends essentially on his genetic characteristics.

The second mode, cognitive, is slower and requires the intervention of different brain structures, especially the prefrontal cortex (Fuster, 1989), and of neuromodulators such as noradrenaline, serotonin and dopamine. This mode makes possible to retain information long enough to have access to consciousness. Briefly, noradrenaline and serotonin act upstream from dopamine. Noradrenaline is in immediate contact with external stimuli and increases the ratio signal/noise whereas serotonin decreases this ratio, likely to protect the central nervous system from unexpected intrusions (Aghajanian et al., 1990; Sara et al., 1994). Finally, the dopaminergic neurons define a functional hierarchy between cortical and subcortical structures in order to respond adequately to the entering information. The main step of the cognitive mode is the working memory which is under the control of the dopaminergic innervation of the prefrontal cortex and its D1 receptors (Sawaguchi and Goldman-Rakic, 1991; Murphy et al., 1996). It is at this level of processing that the environment intervenes. For cognitive processing to occur, adequate environmental conditions are necessary; a lack of motivation, or too much stress, are handicaps for cognitive processing. The fact that cognitive processing allows information to be retained for sufficiently long periods of time (Goldman-Rakic, 2002; Edelman and Gally, 2013) means that there can be an association between two events separated in time. It is cognitive processing that gives us access to time.

It is tempting to compare these two modes of functioning with the primary and secondary processes defined by Freud. Similarities clearly exist, such as levels of consciousness or temporality, but differences must not be overlooked. Indeed, primary process is considered as being driven by a principle of pleasure whereas the analogical mode is defined here as the neuronal interactions which take place following external perceptions whatever the target. Similarly, the transformation of analogical mode into cognitive one is the result of an incoherence between the information stored analogically and the entering one rather than the linkage of energy performed by the secondary process.

Two complementary points deserved to be emphasized: first, at birth and in infants, the prefrontal cortex is not yet mature and the neuromodulatory fibers have not all reached their targets; newborn infants only function therefore in the analogical mode; second, during sleep, and particularly during paradoxical sleep, two of these neuromodulators, noradrenalin and serotonin, are inactive. So the brain only functions in the analogical mode during these phases of sleep. As a dream can only exist insofar as it reaches consciousness, we cannot dream during paradoxical sleep. In adults, the dream is the cognitive expression of the analogical processing that took place during sleep. This cognitive processing is the consequence of the sudden activation of the neuromodulators during phases of micro-awakening which occur about ten times during the night of a good sleeper. The dream, which is a phenomenon of the cognitive kind, therefore brings us closer to the analogical mode, and its possible incoherence stems from the fact that the transition from the analogical mode to the cognitive mode occurs within the space of some tens or hundreds of milliseconds, which means it cannot be confronted with reality.

An example, precisely in a playful form, will help to illustrate the oscillations that occur between analogical and cognitive processing in the waking state. First possibility: “Let’s suppose that I guarantee you a very big reward if you can tell me tomorrow how many people there are in this room today.” Second possibility: “Let’s suppose that I guarantee you this very large reward if you can tell me tomorrow how many people there were in this room today” (that is yesterday rather than tomorrow). In the first case, you are going to count all the people in this room today. You will process things cognitively and you will come up, for example, with 283 people. You will store this figure, 283, in the analogical mode, because it is not possible to store anything for very long cognitively. In so doing, you will lose some information, the number of women and men, of young people and old people, etc.
The following day, when I ask you how many people there were in this room, you will reactivate cognitively the 283 that you had stored analogically, and it is very likely that your answer will be exact. Now, if I ask you the question tomorrow without warning you beforehand that I am going to ask you this question, you will call on your analogical memory of today and you will reconstruct from this memory a probable number (number of rows, number of people per row). You will not answer 50, but nor will you say 1000. Your figure will be in-between, probably inexact, but plausible, between 200 and 350. In this case, with the exception of the organizers of the congress who had counted the participants, you did not count them and your analogical memory never followed the cognitive mode, contrary to those in the group to whom I had put the question on the day itself and who had counted the number of participants. The analogical response is likely to be numerically inexact because the analogical mode does not count precisely beyond a certain threshold. On the other hand, the analogical mode that included the cognitive mode may be exact. In this last case, the basin of attraction, which we will define further on, is combined with the cognitive mode.

Cognitive processing could only occur because there was a motivation that activated the neuromodulators (the promised reward created a motivation). If the absence of a correct response had been associated with a vital risk, it is probable that the number of errors in the counting would have increased, because cognitive processing follows an inverted U curve; without motivation (or without stress, it does not take place, and in the presence of too much motivation (and thus stress, it gets blocked). We can see, then, how much the environment can intervene in cognitive processing and, ultimately, in psychological development. The oscillations between cognitive and analogical processing seem vitally important for understanding the functioning of the central nervous system.

Another element deserves to be emphasized in the case that concerns us: as we have said, at birth, neuromodulators and the prefrontal cortex are not yet functional and the first contacts of the baby with its environment are essentially analogical. At the age of 5 or 6, at a time when cognitive processing has gradually developed (it seems that it appears around the second year of life), a large portion of memories and learning experiences have bypassed the cognitive mode and are thus very difficult to extract cognitively. A child of 6 or even an adult would have difficulty describing how he learnt to walk, i.e., how he built his procedural memory. This is the notion of infantile amnesia, which is not an absence of learning but learning that is difficult to reconstruct at the cognitive level.

And this is probably one of the difficulties that psychoanalysis faces: it may potentially find a certain number of analogical elements that were established during a period when cognitive processing existed, but it becomes more difficult – which does not mean that it is impossible – to go back over the strata until a period when cognitive processing did not exist. The interpretation of free associations is liable to be much more complex when regression reaches this level. It is unlikely that a single interpretation will be sufficient, by itself, to find the origin of a malaise, except perhaps in a very young child.

Let us try to see now how analogical processing is established and memorized. The brain is made up of about 85 billion neurons which, roughly, are all interconnected, directly or indirectly. When several neurons are activated by the same stimulus, connections are established and reinforced to the extent that the experience is reproduced identically; on the contrary, when the experience differs, the connections are modified. This system of memorization makes it possible to recognize instantaneously a visual, auditory or tactile stimulus without it being necessary to analyze it.

Memories are stored according to “Hebb’s (1961) rule” (1948): when two neurons are activated simultaneously during the input of information, the force of their linking, called “synaptic weight,” increases. Conversely, when the activity of one of the neurons increases while that of the other decreases, the synaptic weight linking them diminishes. It is the repeated input of the same information that gives birth to a memory, the quantity of information being limited only by the number of neurons in the system. Once the memory has been registered, any part of the memory suffices to make the system converge toward a stable state: the memory is restored in its totality. According to Hopfield (1982), each memory unit corresponds to a minimal state of energy, a state described as a basin of attraction. This basin “attracts” toward a common memory information that has been acquired simultaneously (certain components of a face or events that took place at the same time). This last point is particularly important because it means that analogical processing is not only fast, as we have already said, because perceptions reach the networks in less than 60 ms, but also simultaneous because the associations made between two perceptions will not take place if they occur at different points in time. This system is thus very efficient concerning its speed and the possibility that it gives the central nervous system of processing several events in parallel, but it is also particularly sensitive to “errors.” Indeed, insofar as the totality of a memory can be recruited from a few elements, two distinct stimuli, but which possess some common elements of a memory, could be considered as identical. These errors will obviously play a role in the content of our memories. It is at this level that cognitive processing takes on its full importance: it makes it possible to correct a part of these errors and, in return, to modify the basins of attraction at the origin of these errors.

The two modes of processing and the oscillations between these two modes are thus indispensable for psychic development. Play may thus be seen as one of the elements that facilitates this development: indeed, if we consider the “pure” analogical mode, that is, the mode that consists only of basins of attraction unblended with the cognitive mode and which only processes perceptions, whether they are sensory or affective, then play helps to give them a reality, and consequently gives them access to cognitive processing. This is just as true for the wooden reel game in which the child is accessing his first cognitive processes as it is for games of “make-believe” or for the doctor games of older children. Two elements, at least, facilitate this transition: on the one hand, the existence of repetitions which form and consolidate the analogical basins of attraction and, on the other, the pleasure obtained from play (repetition is a source of pleasure in the child), which stimulates the modulators that are indispensable for cognitive processing (Tassin, 1998; Tassin and Tisseron, 2014). Having access to the cognitive mode creates the possibility of symbolizing, elaborating and depicting an event. We can see clearly, then, the interest of play in this context.

Finally, we come to the transference. According to Laplanche and Pontalis (1967) [1973]), the transference in psychoanalysis denotes “*a process of actualization of unconscious wishes. Transference uses specific objects and operates in the framework of a specific relationship established with these objects*… *In the transference, infantile prototypes re-emerge and are experienced with a strong sensation of immediacy*” (p. 455). This definition needs to be slightly modified, of course, in the context of children. Nevertheless, transference also exists in the child insofar as, for a neurobiologist, the transference corresponds to the almost- inevitable response of a brain that has no precise information about the objects, in the psychoanalytic sense of the word, that surround it. As we described earlier, one element is enough to stimulate the entire basin of attraction and thus concerns all the elements that gave rise to this basin, including, and especially, those that are not directly related to the object, but which contain particularly early and intense affects. These analogical errors are an important source of information; they are also easily accepted because they do not represent a danger in reality. This reminds us of how play can reactivate, without intrusion, forms of memory that are barely accessible.

To sum up, neurobiological hypotheses confirm observations that play is an important factor in the development of the young child. It is present in all young mammals; in animals, it generally seems to participate in motor activities. The play of infants plays a role in stimulating brain functioning and its development by facilitating the oscillation between two modes of brain functioning, the analogical mode and the cognitive mode. Play facilitates the oscillation between these two modes of functioning which are indispensable for psychic functioning. Two elements present in play underpin this process: the existence of repetitions that form and consolidate the analogical basins of attraction, and the pleasure provided by play which stimulates the neuromodulators that are indispensable for cognitive processing. In psychopathology, play helps the therapist not only to have access to the analogical basins of the young patient, but also to do so without being intrusive, which, in certain cases, may lead to defensive reactions that are difficult to reverse.

But how can we make use of this model of play in artistic therapeutic mediation settings and show its pertinence in the therapeutic process?

## Methodology: Transposition of the Model of Play to Therapeutic Mediations

The utilization of play as a model for evaluating the processes at work in therapeutic mediations organized around an artistic medium makes it possible to show precisely the complexity of the capacities for symbolization acquired during treatment. The model of play will, in fact, make it possible to grapple with what has remained outside symbolization, models of communication based on affect, the language of the body and of the act. This perspective extends in a new way earlier studies on artistic mediations based on the use of a pliable medium which provide the foundations for a general theory of mediation and of its place in the process of symbolization,^2^ that is, a theory of the key issues mobilized in a clinical encounter based on the processes of change initiated by the proposition of a medium. These studies are based on the hypothesis that mediation settings make it possible to engage specific processes of symbolization by bringing into play the sensoriality and motor functioning of the subjects confronted with a medium. Brun et al. (2013) have shown the specificity of symbolization in relation to sensory-motor associativity and the register of gestures, mimicry and postures in therapeutic mediations. The starting point of the process is the encounter with the medium which initiates the emergence of primary forms of symbolization in connection with primitive modes of communication between the baby and his or her environment, in particular, auto-subjective or intersubjective games, which are thus the basis of interrelationships with others.

One of the original aspects of these recent studies consists, then, in placing the accent on sensorimotor forms of language and affective/bodily associativity, as well as the issue of intersubjectivity at work in therapeutic mediation settings (Brun, 2019a). The leitmotif of this article consists in showing that these settings allow in particular for a resumption of the games which historically did not take place, because they mobilize, precisely, sensory-motor associativity in relation to the pliable medium. These settings thus appear to be particularly adapted to clinical configurations that cannot function sufficiently according to the pleasure principle, but which are in a logic of survival, as is the case, for example, with psychotic states of mind or with the psychopathology of violent acting out. These clinical situations cannot be approached in terms of the traditional model of the dream, with recourse to a work of symbolization based on the absence of the object and on a suspension of motor functioning and perception. On the contrary, it is a matter of promoting with these patients certain forms of symbolization that help to transform traumatic experiences into a certain amount of pleasure. The specificity of the therapeutic work consists in rediscovering the traces of the potential play that degenerated in the history of relations between the infant and his or her environment, and to create the conditions that are necessary for regenerating this potential play which will then be able to deploy its symbolizing potentialities. In this process of reviving play, play can only be revived if it is “between-play” (*entre-jeu*), if it is *jeu à deux*, thus only if the clinician recognizes, accepts and shares the potential play. In other words, it is not just a matter of identifying the traces of the potential play that could not be deployed, but it is necessary to carry out a work of reconstruction to establish what historically, in the responses of the environment, prevented this potential from being deployed. This work of reconstruction is accomplished in particular by deploying different forms of play in therapeutic mediations.

A modelisation based on the model of play, as presented in this article, takes into account the question of the relations between the manifest aspects of play and its latent issues, the evaluation of the latter implying a theory of symbolization based on what is involved in the play. Taking as our starting-point the identification by R. Roussillon of the interlocking of different forms of play, we have developed, for example, the identification of a series of “interlocking” games of presence/absence whose later forms appear to be more developed and complex forms of the former: peek-a-boo games, games of throwing and bringing back (spatula game), game of the wooden reel, mirror games, games of “going to war,” etc. This example of the interlocking of a series of games offers a model for the work accomplished by the authors of this article to make the tool pertinent: it is a matter of trying to identify the series of games that are interrelated and that represent forms of a fundamental psychic issue that becomes increasingly complex over the years. The interest of this attempt to offer a modelisation is that it makes it possible to follow the same basic issue as it is progressively elaborated over the course of time, and to identify the typical forms that follow each other in this elaboration. It also makes it possible to evaluate the degree of psychic complexity acquired by the issue in question. From one form of play to another we can thus evaluate the change, or the progression, of the psychic elaboration.

The model of play makes it possible to take up again what has remained outside symbolization and models of communication based on affect, the language of the body and of the act. It is a matter of promoting certain forms of symbolization that help to transform traumatic experiences into a certain degree of pleasure.

The number of “typical games” that can be enumerated is significant and has its place within a temporality and historicity. The “typical games” that children enjoy, even if they present invariant forms over the course of life, are not in fact the same or do not take on the same forms during the course of the subject’s development and of the degree of increasing complexity of psychic life. This research proposes to draw on a model for evaluating processes of symbolization, based on play, in order to elaborate a modelisation of the evaluation of modes of symbolization beyond therapeutic processes. A clinical example will now be developed with aim of trying to understand the “logics” of play (Brun, 2019b) at work in a therapeutic process assisted by therapeutic mediations, in this case pictorial mediation.

## A Clinical Example of Qualitative Evaluation Based on the Play of Therapeutic Mediations

Therapeutic mediations with psychotic children make it possible for the uncompleted process of games shared with the primary environment to be resumed: the initiation or reinitiation of sensory-motor games shared with therapists reintroduces the potentiality of a sensory-motor symbolization. The initiation of games is situated at the level of the primitive sensorial experiences aroused directly by the sensoriality of the pliable medium, such as painting, earth, water, collage, etc., which is always, as Milner (1955) conceptualized it, both the mediating object, the material, and the therapist presenting the medium in its materiality, such as painting, earth, water, collage, etc.

**TABLE 1:** Table of games based on the clinical example.

	Games sensory-motor exploration	Mirror games	Games shared with the group
	*Multisensory games*	*Intersubjective games*	*Intersubjective and intrasubjective games*
			*Reflexivity*
	- sensory-motor games of discovery	- Sound, visual, tactile mirror games	- Games of hide-and- seek
	- Games with sensory- motor forms	- Chase games	- Circle games
		- Peekaboo games	- Make-believe games
	- Games of destroyed/found (R. Roussillon)	- Games of sensory transpositions	- Participation in group associative chains
	- Games of gathering together and dispersion	- Games of tuning	- Collective narrative games
		- Theatralisation	- Games with fantasy scenarios
	**MATTER**	**MATTER**	**MATTER**
**Game with hallucinated sensations : Establishment of primary forms of symbolisation**	Non transformable	Transformability of the states of matter	I actor and subject of the transformations of matter
	**FORM**	**FORM**	**FORM**
	Irreversibility and destruction of the form	Reversibility of the transformation of the forms	Representative forms with a figurative content
	**The child**	**The child**	**The child**
	*It is erased it disappears it liquefies a support collapses* **Process without subject (R. Roussillon)**	*A door opens and closes It appears, disappears and reappears, it folds and unfolds*	*Figurative representations in his painting*

But first, how do we identify “the traces of games that, historically, could not take place”? Here is the example of a therapeutic painting group for young psychotic and autistic children in an institutional setting to help us identify a logic of the organizers of games in the group: we will focus here on just one child who was chosen precisely on account of his primary impossibility of using the setting to play.

He was presented as a very violent child, in a symbiotic psychosis, and inseparable from his mother. He is being cared for in a pictorial mediation group in a Medical and Psychological Consultation Centre (CMP) run by a couple consisting of a psychologist and a nurse. A female clinical psychologist^3^ and psychoanalyst has the function in this group of a writing observer, whose task is to observe the language of the body and of acts, that is to say, sensory- motor associativity.

### Games of Sensory-Motor Exploration

During his first therapeutic group of painting, the child at the beginning of latency-age, screamed and struggled as soon as the door was closed; above all, he hit the nurse, but also everything that was within his reach, and tore off sheets of paper stuck on the wall. The therapists had to restrain him on several occasions to stop him from hitting the other children. As soon as he was no longer contained by an adult, he would throw himself violently on the floor or begin hitting again. If he was presented with a sheet of paper, he tore it up. In the second group, his entrance into the workshop was equally violent, and this gradually became unliveable, intolerable.
The psychologist/therapist then had the idea of sticking back on the wall the sheet of paper he had torn off, while evoking his fear that his companion would disappear from the waiting-room and that everything in the workshop would be torn to pieces. From her place as the writing observer, the psychologist also talked to him about his fear that the therapists might become as nasty as he told the group they were, about his terror that we (the therapists) would shut him away or hit him. The child tore the patched up sheet of paper off the wall again; the therapist stuck it back on calmly and then an alternation began between the child’s activity of tearing and the therapist’s patching up of the shreds. In the following groups, a sort of rhythm was established between the psychologist and the child, who tore off the sheet of paper more and more gently and waited until the therapist had stuck it back again before tearing it off once more…

The child was inflicting on the sheet of paper his own terror of being torn to pieces, violently penetrated, in a process of active/passive reversal. An important process then appeared, as the groups continued: this child’s physical attacks began to diminish when they were transposed onto the medium of painting, thanks to the sensory-motor response of the therapist. The child had met a therapist who could resist and survive his destructiveness, so he was now able to destroy/find the therapist in the game that the psychologist was playing with him and destroy/find the sheet of paper: therapist and mediating object are inseparable in the process of destroyed/found (Roussillon, 1991).

Nevertheless, he would often let himself fall headlong, sometimes banging his head on the ground, or, at certain moments he would flow, literally like water, at the foot of the war and try to leave the room, always in a terrifying state of panic. The therapists exhausted themselves trying to find strategies of spatial organization to control him.

Let us try to give some meaning to his acts. The fact that the child threw himself on the ground when he was released by the therapists who were containing him physically shows that he is afraid of being “let down,” dropped, and thrown into the void, a terror that also manifests itself in another way when, having become all soft, he flows, like water, on the ground, which suggests terrors of breakdown, perhaps of liquefaction. We also noticed that these breakdowns occurred when he stopped acting violently. We can make the hypothesis that one of the functions of these repeated physical attacks consists in clinging, as it were, to motor functioning and to the movements of attack, which have a self-containing function as well as a function of gathering together a subject confronted with strong feelings of terror of fragmentation.

How are we to understand that he goes as far as to hurt himself by banging his head on the ground? These physical self-attacks correspond to forms of paradoxical defense described by Roussillon (1991). The child enacts and inflicts on himself his terrors rather than suffering them passively. It is a technique of survival; he is so terrified that the unknown therapists will drop him, let him down, that he prefers to inflict these traumatic experiences on himself rather than risk being subjected to them by potentially persecuting therapists. In short, he is portraying his primitive terrors, so it is necessary to decipher his body language and interpret his way of portraying his terrors. This is necessary in order to understand the processes of desymbolization involved and to be able to offer the child suitable responses.

The necessary precondition for re-establishing games that were not played and for the emergence of the symbolization that accompanies these games consists therefore in being able to symbolize the desymbolisation: it is necessary first to symbolize the traumas that had thwarted the processes of symbolization.^4^

### Mirror Games

The child gradually entered into an exclusive mirror relationship with the therapist, a mirror relationship…

To accompany the child in his troubles to leave his companion, we decided to put the painting sheet in front of the closed door. The child, who was initially in the corridor glued to his companion, accepted to stay in a room and gradually took part in another game with the psychologist: he began by walking on the sheet of paper, whereupon the psychologist pretended to move forward, which made the child withdraw in a hurry, introducing a game of the kind “catch me if you can.” Here he hid behind a piece of furniture and reappeared again, with the therapist exclaiming, “Ah, there you are!” and so a peek-a-boo game began. The child gradually agreed to let the therapist draw the contour of his feet on the sheet of paper, then that of his hands. A gathering together of his body on the sheet of paper occurred, first with bits of hand and feet, then the psychologist drew the child’s whole silhouette on a sheet of paper, a painting that the child contemplated attentively, and then with joy. The child subsequently began to paint, on the floor and then on the wall right next to the door, and, finally, it got easier for him to be part of the group, leaving his companion outside.

What appears here, then, is a chase game, “catch me, if you can,” and a game of peek-a-boo initiated by the child, and a game of gathering together on the sheet of paper, initiated by the therapist, with the inventiveness of the therapist who catches the child by gathering together bits of his body, the feet and the hands surrounded with paint, which corresponds absolutely to the fragmented bodily experience of this child.

The first games in the group for these children who do not know how to play are therefore first of all games introduced by the therapist, mirror games, which are necessary before the child can meet the group of the other children and enter into group games. These initial games are based on the sensations of the medium and it is the sensorial attraction of the medium explored with a therapist that initiates the transference process onto the medium. Games of sensory exploration belong to the most archaic typical games for every baby, but what characterizes clinical situations where playing is a matter of difficulty is either the absence of sensory exploration, with withdrawal, as in severe forms of autism, or explorations with a lot of destructiveness. The role played by sensory-motor functioning is envisaged here in terms of the modalities of relating to the environment, just as the emergence of sensory-motor forms in the work of mediation is always understood in connection with the interactions with the therapists and the group of children. The basic hypothesis underlying this therapeutic work involving mediations is that the interpretation of the child’s behavior and of his or her singular relationship to the pliable medium harks back to the account of his or her primary interactions (Brun et al., 2013).

This sequence with the child was fundamental because our bodily, but also verbal interventions, put the emphasis not on the destructiveness of the child but on our involvement in, and accompaniment of, his exploration of the medium. We attributed to him the intention of exploring the material setting that we shared with him. It is thus the therapist’s response that enables the child to see himself as having this or that intention. Based on the interpretation given to him of his acts, the child, insofar as he is experienced as being destructive, will identify himself with this intention of destructiveness, whereas the child who is experienced as an explorer will, on the contrary, identify himself with this intention of potential creativity.

### Shared Games With the Group

Next, the child joined the group of children where the therapists propose games of sensory transposition: for example, they associate group movements with sound rhythms and, sometimes, the whole group paints at the same rhythm, with singing. These games of bodily and sensory-affective tuning re-establish a body mirror through the tuning of the rhythms, postures, gestures, facial expressions and exchanges of sounds, accompanied by emotional exchanges, aimed at re-establishing the mirror function of the primary relationship. Enacted interpretations or “interpretactions” (Prat, 2014), based on sensory-motor associativity, are predominant here.

The child gradually took part in group games, peek-a-boo games, circle games, for example, lining up in a row to form a train, chase games, make-believe games in which, for example, the group plays at imitating animals, lions, dogs, wolves, etc. He took part in collective narrative games, for example, playing at being the baby.

He also introduced games with fantasy scenarios.

### Games of Formal Associativity

After the first phase of attack and tearing of the sheets of paper, the child then set about diluting the traces of paint that he had made on his sheet of paper with water until they were completely erased and often until the wet and curled sheet of paper had been perforated.

Here, we can see again an initial form of an inner resumption of primitive terrors, like the terror of erasure, disappearing or dissolution, or of breakdown, reflecting what the child does to the sheet of paper. He is, so to speak, in a mirror relationship with the sheet of paper for painting, “a perforated, pierced or torn sheet of paper/skin,” “an I/paint that liquefies,” which disappears or which is sucked into a hole, “a form that is erased” or again “a support that collapses.”

We have seen that the sensations which, in reality, are furnished by the materiality of the medium, reactualize hallucinated sensations in connection with terrifying primitive experiences.

After 15 months of group, the child began to draw keys and doors which he cut up and stuck on the wall with a hinge for opening and closing them. He played at hiding things like scissors behind his paper door, which he opened and closed while laughing. This is no longer the figure of a formal signifier of torn off skin or of a destruction of form, but of “a door opening and closing; it appears, it disappears and it reappears; or, it can be folded or unfolded.” He can play the wooden reel game with the key, make the object key disappear and reappear; the child’s physical attacks with the group had stopped.
Fantasy-based games with scenarios then appeared in the child and in the group as a whole.

It is thus the transference of the sensory-motor functioning of the children onto a sensory medium, represented by the clinicians, the mediating material and the setting, with the responses of the clinicians through play, in sensory-motor as well as verbal language, which helps the children to evolve from a dismantled (Meltzer et al., 1975) and destructive sensoriality toward a sensory recomposition, to a “*mantèlement*” or re-organization of the sensory-motor register in the relationship with the pliable medium, with a work of gathering together the fragmented islets of the child’s ego in psychotic and autistic configurations.

The role played by games in these situations has echoes in anthropology: if we situate the interest of anthropology for play at the level of the organization or reorganization of scattered or “orphaned” sensations, the beginnings or fragments of acts or gestures, the parallel can be made with the games of “societies.” It is thus possible to highlight an anthropological dimension of play which is inseparably individual and collective. When the Aztecs practice or watch their ball game, they symbolize the path of the sun and the latex ball reproduces, as it bounces, the movement of the stars which must periodically be stimulated to avoid entropy. They not only enact and ritualize an inevitable loss of energy and the bloody sacrifice that is alone capable of circumvening and guaranteeing the continuity of the cosmological movement; through this game, the Aztecs, live, think and metaphorize the osmosis between the life of individuals and that of the cosmos. Analogically speaking, it is a way of matching individual passions and forms of collective functioning. The same thing could be said of the simultaneous emergence of ball games in England in the eighteenth century and of the parliamentary regime (Elias and Dunning, 1994): it is the way a given society chooses to metabolize violence by proposing forms of play that are nourished by conflicts and tensions while regulating them. These games can be called social in the sense that they are based on collective practices at the scale of a society. But we must remember that Marcel Mauss considered anthropology as the discipline interested in a human being: all at once physiological, psychological and social. These three dimensions do not overlap but act in concert. These games, such as the tlachtli practiced by the Aztecs during the colonial era (see Duverger, 1979), make it possible to learn, through the body, the tendencies of a society. Ollin is the nahuatl name of movement and also of the ball and signifies as much the movement of the player as that of the sun. Thus, by playing and watching the game, everyone takes on the dimension of their inscription in the world and learns gestures “ecologisation,” of sensations and feelings. It is through these play practices that the interior and exterior dimensions of the human body are inscribed in a certain form of operating continuity. Energy must be maintained to compensate for its exhaustion; as such, it becomes the source of a discipline of life, continually remembered and shared, which relates inseparably to all forms of movement.

To return to the analysis based on the case of the child, the reactualization of experiences in the register of primitive agonies, in the form of hallucinated sensations, which take shape, often in a playful way, in primary forms of symbolization, evidences the primordial role played by hallucination therapeutic mediations. These processes hark back to Freud’s second theory of hallucination in “Constructions in analysis” (1937), where he describes the possible hallucinatory return in analytic treatment of “something that a child has seen or heard at a time when he could still hardly speak” (p. 267). This is an epistemological revolution because hallucination is no longer a wish-fulfilment, as in dreams, but turns out to be made up of unsymbolized sensory-perceptual elements that have not been transformed into images or words. Freud thus describes a process in which the distinction between psychic registration and external perception is erased. In other words, these hallucinatory experiences correspond to a mode of hallucinatory return of traumatic events or modes of relationship that were once perceived but that are mixed up and confused with the present. They are experiences that cannot be recalled because they cannot constitute themselves as memories, but they can return in hallucinations, in the form of perceptual traces that are often linked to bodily states and sensations. These perceptual traces are unrepresentable, unsubjectivized and not appropriated. It is not a matter, therefore, of rediscovering a latent meaning or a lost representation, but rather of bringng back into play chaotic, ungraspable and incomplete representative processes. At the end of Freud’s work, the question of subjective appropriation no longer concerns only the issue of becoming conscious (1923) but, with the introduction of splitting, the way in which certain aspects of the psyche are not represented, involving different forms of psychic withdrawal: this path was to be explored by Winnicott (1974) with his conceptualization of primitive agonies, experiences that are so catastrophic that the subject, he writes, “withdraws from his subjectivity.” The subject thus withdraws from himself, cuts himself off from his subjective experience to avoid experiencing extreme suffering, without any solution, without representation, and without end. Primitive agonies, as we saw earlier, are terrors of liquefaction, fragmentation, annihilation and disintegration that would annihilate the subject if he experienced them; so he withdraws from himself in order to survive: this is why Winnicott writes that these experiences have not yet occurred, have not yet been experienced, and have not yet been integrated with psychic life. These unintegrated traumatic experiences, which Bion calls experiences of “nameless dread” will also reappear in the language of the act and of the body, that is, in the forms of language concomitant with their registration in the psyche, before the acquisition of verbal language, according to Freud. This is not a process that pertains to repression but rather to a form of splitting: what is involved is a real amputation of the subject who is unable to master his traumatic experience, his catastrophe of identity. Certain aspects of psychic life are neither represented nor integrated in the fabric of subjectivity and they mainly concern the question of identity. To be more precise, they undermine the subjectivizing function of the ego, in other words the process of subjective appropriation. This essential attack on the reflexive function affects, according to Roussillon’s (2008) formulation the capacity to see oneself, to hear oneself and to feel.

But these split-off experiences, these experiences of subjective withdrawal, will not cease to return; they are subject to the repetition compulsion in the hope of an eventual subjective integration, and this return will pose a threat for the solutions of psychic life of the subject who has set up defense mechanisms precisely in order to fend against the return of these split-off experiences. Roussillon (1991) has called these defenses against primary traumatic experiences that provoked an experience of psychic death “paradoxical defenses”: affective coldness, disorganization of the links of cognition, in Bionian terms, attacks of linking on thinking, are envisaged as effects of the incapacity to feel and to be in touch with oneself, to the extent that they seem to be reactional to the return of the traumatic history of attempts at symbolization. Moreover, primitive terrors will self-represent their forms of withdrawal: for example, the subject withdraws by disintegrating or liquefying himself, by immobilizing parts of himself or by freezing his affects: identifying these processes will constitute a therapeutic lever, as we have highlighted in the clinical work with the child.

Finally, this clinical example shows how interpretative modalities based on play permit the emergence of primary forms of symbolization: it is necessary to rediscover the traces of games that are indispensable for the development of symbolization, typical games that could not take place historically during the infant’s life: it is this process of reactivating games that have not yet been played that enables the children to gain access to the first forms of symbolization. Hallucination thus plays an essential role in these processes mobilized by games, within mediation settings, in the form of a reactualization of hallucinated sensations which will acquire shape, through the activation of typical games, in the pliable medium. This shaping occurs in the material with the emergence of sensory-motor forms, and also in the games as a whole, which we have called multisensory games, games of sensory-motor exploration, intersubjective games, mirror games, and intrasubjective games accompanied by the acquisition of a reflexive capacity, with shared group games. This emergence of sensory- motor forms in the work of mediation and during the activation of typical games is always understood in connection with the interactions with the therapists and the group of children. One of our fundamental hypotheses is that the interpretation of the child’s behavior and of his singular relationship with the pliable medium refers back to the account of his first interactions.

In this specific therapeutic work centerd around the activation of typical games, which permits the emergence of primary forms of symbolization, it is a matter both of interpreting the modalities of the transference constellation (Freud) on to the pliable medium with children in great psychic suffering, in the sense of transference on to the material, the therapist, and transference on to the setting, and of describing in this context the specific interpretative modalities of the therapists, who make extensive use, as we have already said, of body language, facial expressions and gestures, as well as theatralization, all ways of activating the games of early childhood or childhood. The clinical example also helps to show how it is possible both to interpret a transference onto the sensory-motor forms of the pliable medium and to interpret these sensory-motor forms as manifestations of the transference.

Therapeutic mediations thus permit the initiation or re-initiation of sensory-motor games shared with the therapists, thereby reintroducing the potentiality of a symbolization based on sensory-motor associativity.

Table 1 transcribes a qualitative evaluation of the logics of the games (re)established in the therapeutic work with the child.

## Inventory of Typical Games in Therapeutic Mediation Settings

Based on the observation of typical games, depending on the ages and pathologies of the children, in therapeutic mediation settings, a table identifying the presence of the principal typical games in therapeutic mediations has been drawn up, in a transversal dimension, with the countries participating in the research, France, Brazil, Canada, Italy, and Belgium. There are no apparent significant differences between countries, but differences can be observed over the course of time and depending on the pathologies. In Brazil, Vieira and Zornig (2015) and Zornig (2015, 2019) points out how much children, whose life history is potentially traumatizing, need a first stage of primary isolation that can help them to play with their sensations, before engaging in intersubjective play. The “position of active passivity of the analyst,” to take up.

**TABLE 2:** Typical games and therapeutic mediations Typical games.

**Baby games. 0–12 months (based on Guerra, 2019). Crèche *observation***
(1) Eye catching games (Bodily support). (0–2 m.)
(2) Games of protoconversations (Face to face games). (2 m.)
(3) Games of imitation
(4) Games of tickling and suspense (3–5 m.)
(5) Vocal games (5–12 m.)
(6) Games of displacement in space with a referential gaze (5–7 m.)
(8) Game of hide-and-seek (8 m.)
(9) Games of affective tuning. (9–12 m.)
(10) Games of interlucidity. (8–12 m.)

**Spontaneous game of multi-sensory exploration**
Spontaneous free activity (Pikler, 1979)
Sensory-motor games of discovery (Brun et al., 2016; Brun, 2019b)
Games with sensory-motor forms (Brun et al., 2016; Brun, 2019b)
Sensory-motor exploration of the object presented. (*object presenting)* (Winnicott, 1971)
Games of free discovery of the sensory qualities of the pliable medium (Milner, 1955; Roussillon, 2013)

**Game of gathering together and dispersion**
Game of construction, interlockings and collapse (Roussillon, 2008)
Game of a train of children with a possibility of derailment. (Jacquet, 2012)

**Jeux de présence-absence (Roussillon, 1991, 1995, 1999, 2008, 2019)**
Game of throwing away/bringing back by another person
Spatula game described by Winnicott (1941)
Game of “peekaboo”: finding the other person and seeing oneself found by the other person.
Game of hide-and-seek
Wooden reel game described by Freud (1937)

**Tuning games**
Rhythmic, tonic-postural tunings (Roussillon, 2008; Stern, 1985)

**Mirror games**
Mirror stage (Lacan, 1953)
Mime mirror game
Immediate imitation behaviors of the other (Stern, 1985)
Choreographed displacements in space
Sound, visual and tactile mirrors
Circle game
Orchestra conductor game

**Games of seduction and/or of seduction-fascination**
Games with a perverse coloring (Ravit, 2016, 2019)
Games with death and radical negativity (Ravit, 2016, 2019)

Roussillon’s (1991) formulation, who “plays at not playing,” seems to be fundamental if the child is to be able to explore the analytic context without feeling invaded or threatened, and to recognize in the analyst an object that “cushions” the disruptive effects of the instinctual drive discharge, so that transitionality can begin to be established between the destruction of the object and its creation. In Italy, Zurlo (2017) has not noted significant differences between countries in the practice of therapeutic mediations.

Generally speaking, the more severe the pathologies are, as in psychoses, the more patients, both children and adults, find it impossible to play, and it is necessary to put back into play many forms of games so that the patients can gain access to the processes of symbolization.

The beginning of this survey concerns the games of babies, from 0 to 12 months, carried out in the context of crèches: this work is inspired by the indicators of intersubjectivity identified by Guerra (2019)^5^. Table 2 lists the games whose modelization has been developed by the authors of this article. This table is obviously not exhaustive and other forms of games must be explored, for example, games of holding (game of throwing/fetching (fathers with babies), games of stability (balancing games, tightrope walking), game of letting go/holding on (game of mutual trust, e.g., “the blind man and his guide”), chase games like the game of the “Big Bad Wolf” or of “playing tag,” betting games like “games of bluff” and “playing forfeits” (described by Freud, 1909. These other typical games are being explored and are in the early stages of modelization.

## Conclusion

The recourse to the model of play in settings of artistic therapeutic mediations makes it possible to reactualize experiences that have not been integrated with subjectivity, both bodily and psychic experiences, often in the register of primitive agonies. This hallucinatory reactualisation supported by typical games proves to be the point of departure for the emergence of primary forms of symbolization in patients who can thereby appropriate unsubjectivised experiences in order to weave them into their subjectivity. This process occurs in part thanks to the resumption of sensory-motor games shared with the clinicians. It is thus a matter of mobilizing chaotic, unthinkable and incomplete representative processes by taking into consideration this emergence of primary forms of symbolization within an intersubjective context. This resumption of the incompleted process of games shared with the primary environment involves the transference of the sensory-motor functioning of the patients on to the pliable medium in its materiality and on to the clinicians. In this therapeutic context, psychoanalytic listening is necessarily polymorphic: it involves the classical notion of listening to the transference on to the clinicians and on to the group, as well as listening to the transference on to the medium which represents an aspect of the transference on to the setting, in the sense of a transference of primitive forms of relating, but also, in a very specific way, – and this has been the aim of this work – listening to the latent and manifest games that are deployed in the therapeutic process, upon which access to symbolization depends. This article only accounts for a part of the research currently in process; other scales of clinical evaluation based on the identification of typical games are being modelised and will be published in a collective work6.

The multidisciplinary approach, at the crossroads of anthropology, criminology, neuroscience and psychoanalytic clinical psychology, shows the interest of the model of play in the evaluation of settings of therapeutic mediations, with a convergence of the different disciplines emphasizing the pertinence of this model of play.

## Data Availability Statement

The datasets generated for this study are available on request to the corresponding author.

## Ethics Statement

Ethical review and approval was not required for the study on human participants in accordance with the local legislation and institutional requirements. Written informed consent for participation was not provided by the participants’ legal guardians/next of kin because this child’s clinic, written by AB, has been done 20 years ago. Unfortunately, at that time, no informed consent has been written. However, according to French law, this study does not fall within the scope of the “Loi Jardé,” and therefore does not require specific approval by an ethics committee. We thus chose to protect patient privacy by hiding information on the patient life and by hiding and changing recognizable details of the sessions. Moreover, the manuscript does not include any anamnesis data that would allow the child to be recognized: it is only about his psychological life and the manifestations of his anxieties within the group. We fully anonymized the manuscript data. More specifically, the name and the age of the child have been removed. There are no references to the sex of the child. We didn’t specify who was accompanying the child to the sessions.

## Author Contributions

AB directs the international research, coordinated the writing, and wrote the clinical parts of the manuscript. LB, AM, and MR wrote the criminology section. DC have written about anthropology. J-PT have written the section on neuroscience. MZ and SZ participated in the writing of the research results. TG has helped analyzing data, writing the methodology, and contributed to finalize the manuscript. VD, SM, LM, JJ, and EJ participated in the conduct of the research and to the data analysis. RR designed the modelization based on the model of play that is extended in an innovative way in this research.

## Conflict of Interest

The authors declare that the research was conducted in the absence of any commercial or financial relationships that could be construed as a potential conflict of interest.

## Footnotes

1 Translator’s note: from Middle French *mantéler* = to fortify, hence the word *démantèlement* (dismantling).

2 *Manuel des médiations thérapeutique*, Paris: Dunod (New edition, 1st edition 2013).

3 One of the authors of this article.

4 On the symbolization of desymbolization, see Brun and Roussillon (2016) (the details are in the references).

5 Several contributors to this worked with Victor Guerra, before his premature death.
